# Suspected Sexual Transmission of Dermatophilosis among Men Who Have Sex with Men, Lyon and Paris, France, 2025–2026

**DOI:** 10.3201/eid3206.260401

**Published:** 2026-06

**Authors:** Matthieu Degreze, François Durupt, Marine Ibranosyan, Anne-Lise Maucotel, Audrey Lapendry, Laurie Gouillon, Matthieu Godinot, Maxime Bonjour

**Affiliations:** Hospices Civils de Lyon, Lyon, Auvergne-Rhône-Alpes, France (M. Degreze, F. Durupt, M. Ibranosyan, A.-L. Maucotel, A. Lapendry, L. Gouillon, M. Godinot, M. Bonjour); Laboratoire de Biométrie et Biologie Évolutive, UMR CNRS 5558, Villeurbanne, France (M. Bonjour)

**Keywords:** dermatophilosis, bacteria, zoonoses, Dermatophilus congolensis, sexually transmitted infections, sexual transmission, sexual and gender minorities, men who have sex with men, skin diseases, France

## Abstract

We report a genomically linked cluster of 9 *Dermatophilus congolensis* cutaneous infections diagnosed within 2 months among men who have sex with men in Lyon and Paris, France, 2025–2026. Genomic similarity and shared sexual exposures strongly suggest interhuman sexual transmission of this zoonotic bacterium.

*Dermatophilus congolensis* is a gram-positive, facultatively anaerobic actinomycete responsible for dermatophilosis, an exudative dermatitis of animals (*1*). The disease predominantly affects cattle, sheep, and horses, mainly in tropical and subtropical climates. It typically manifests as benign, crusting superficial skin lesions but occasionally progresses to extensive disease, sometimes resulting in significant mortality in cattle herds (*2*,*3*). The pathogenesis relies on 2 factors, skin microabrasions and moisture, which activate motile zoospores to penetrate the epidermis (*1*).

Human infections are rare and considered accidental zoonoses. Those infections are classically described in farmers, hunters, veterinarians, or animal riders following direct contact with infected animals (*4**–**10*). The clinical manifestation typically involves nonpruritic, pustular, and crusty lesions. *D. congolensis* is susceptible to β-lactams, macrolides, and tetracyclines. Systematic susceptibility testing is rarely performed in clinical practice. Reported treatments include penicillins, although lesions are often self-limiting. To date, human-to-human transmission has not been documented, and urban cases without reported animal exposure have rarely been described (*11*). We describe a temporally clustered series of human dermatophilosis cases occurring in France among urban men who have sex with men (MSM) without livestock exposure, raising the hypothesis of an alternative mode of transmission.

## The Study

During December 2025–February 2026, a total of 9 men sought care at the sexually transmitted infections (STI) clinics of the University Hospital in Lyon, France, for skin infections that were determined to be caused by *D. congolensis*. All patients were MSM living in urban areas; they were 22–63 years of age (median 50, interquartile range [IQR] 34–59.5 years). None reported occupational exposure to livestock or direct contact with farm animals or horses, although some did report regular or occasional contact with domestic pets (cats or dogs). None reported recent travel to tropical regions.

**Figure 1:**
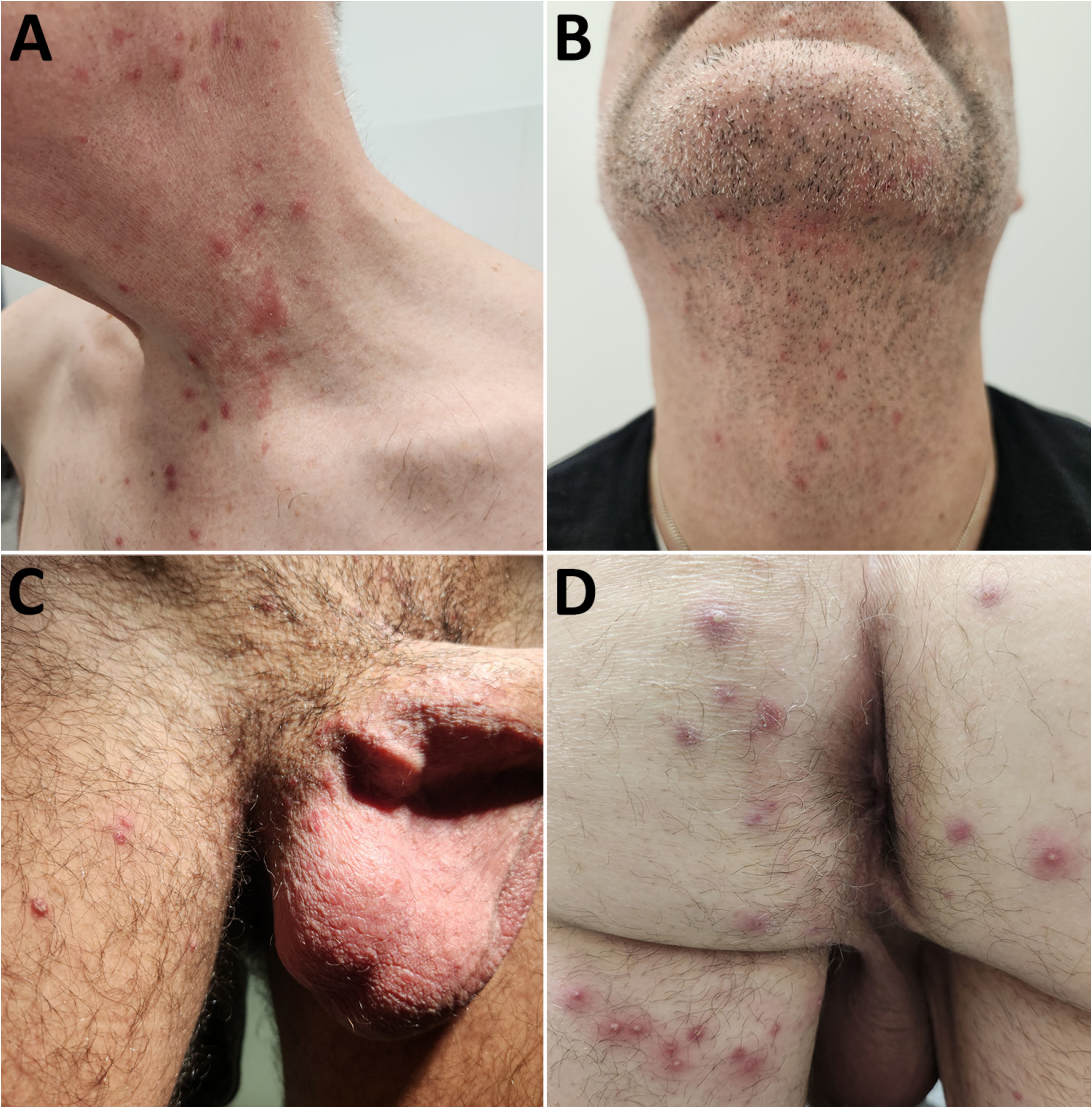
Dermatophilosis lesions in patients in cluster of suspected sexual transmission of dermatophilosis among men who have sex with men, Lyon and Paris, France, 2025–2026. A) Papular lesions of the neck and torso in patient 1. B) Folliculitis of the beard in patient 2. C) Papules of the groin and scrotum in patient 3. D) Papulopustular lesions of the buttocks during second occurrence in patient 1.

All patients exhibited nonspecific erythematous papules, occasionally pustular or squamous, mainly located in the genital region (penis, scrotum, pubic area; n = 8), trunk (n = 5), perioral region (beard area; n = 4), lower limbs (n = 4), and, less frequently, anal margin (n = 1) (Table; Figure 1). Lesions predominantly involved areas exposed during sexual contact, without mucosal involvement. Pruritus was variably present. No systemic symptoms were reported except for patient 7, who experienced fever and vomiting 2 days before medical consultation. Given the clinical manifestations and epidemiologic context, differential diagnoses included staphylococcal folliculitis, sexually transmitted dermatophytosis (*Trichophyton mentagrophytes* genotype VII), *Klebsiella aerogenes* folliculitis, secondary syphilis, mpox, and molluscum contagiosum.

**Table:** Characteristics of 9 patients with cutaneous *Dermatophilus congolensis* infection in cluster of suspected sexual transmission of dermatophilosis among men who have sex with men, Lyon and Paris, France, 2025–2026*

Pt. no.	Age, y	Underlying conditions	Lesion locations	Cutaneous co-infection or superinfection	Sauna attendance (location)	Symptom onset date	STI clinic consultation date	ENA accession nos.
1	63	HIV PrEP use, STI history (CT, HSV)	Neck, beard, trunk, pubic region	Pubic pthiriasis	2–3 times/wk, 2 wks before symptoms (Lyon)	2025 Dec 23	2025 Dec 31	ERS29280943
2	50	HIV PrEP use, STI history (HPV)	Beard	None	2025 Dec 18 (Lyon)	1 week after sauna attendance	2026 Jan 19	ERS29280944
3	26	HIV PrEP use, STI history (CT, NG)	Beard, umbilicus, scrotum, inguinal folds	*Staphylococcus aureus* (beard)	2026 Jan 28 (Lyon); 2026 Feb 1 (Paris)	2026 Feb 1	2026 Feb 6	ERS29314550
4	51	HIV PrEP use, STI history (CT, NG, Tp)	Legs, groin, back, scrotum	None	2026 Feb 3 (Lyon)	2026 Feb 7	2026 Feb 10	ERS29314551
5	60	HIV PrEP use, STI history (Tp)	Penis, pubic region	None	2026 Jan 29–30 (Paris); 2026 Feb 3 (Paris)	2026 Feb 6	2026 Feb 10	ERS29380148
6	59	HIV+ (CD4: 643/mm^3^), STI history (CT, NG, Tp, HSV)	Upper thighs, umbilicus, groin	None	2026 Feb 3 (Lyon)	2026 Feb 11	2026 Feb 18	ERS29380149
7	42	STI history (NG, Tp), cannabis and cocaine use	Scalp, beard, scrotum, abdomen, limbs, anal margin	*S. aureus*	Regular attendance, 1×/wk (Lyon)	2026 Feb 10	2026 Feb 19	ERS29380150
8	45	HIV PrEP use, STI history (CT)	Penis, scrotum, pubic region, upper left thigh	*Staphylococcus lugdunensis*	2026 Feb 5 (Lyon)	2026 Feb 11	2026 Feb 20	ERS29380440
9	22	HIV PrEP use	Scrotum, inguinal folds	*S. aureus*	None	2026 Feb 6	2026 Feb 20	Not sequenced

*CT, *Chlamydia trachomatis*; ENA, European Nucleotide Archive; HSV, herpes simplex virus; HPV, human papillomavirus; NG, *Neisseria gonorrheae*; PrEP, preexposure prophylaxis; Pt., patient; STI, sexual transmitted infection; Tp, *Treponema pallidum* subspecies *pallidum*; +, positive.

**Figure 2:**
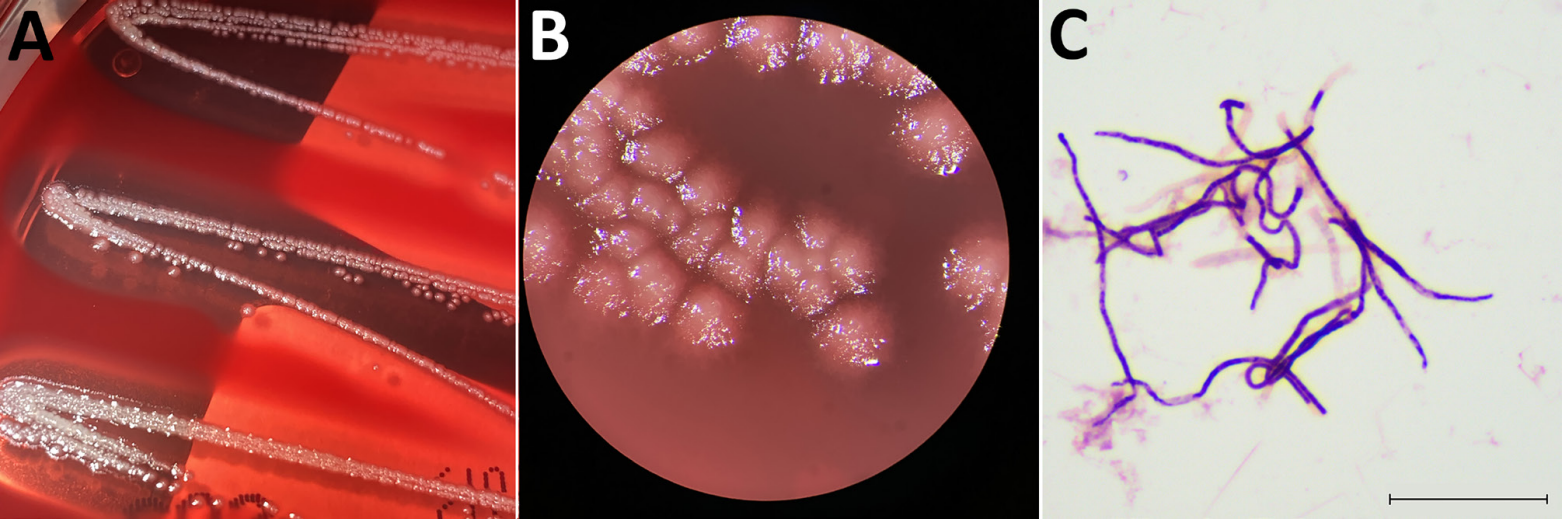
Results of sample testing in cluster of suspected sexual transmission of dermatophilosis among men who have sex with men, Lyon and Paris, France, 2025–2026. A, B) *Dermatophilus congolensis* colonies obtained on blood agar after 40 hours of incubation, seen with the naked eye (A) and by stereomicroscope (B). C) Microscopic examination of *D. congolensis* isolated from patient 1 skin sample. Gram staining; scale bar indicates 25 μm.

For all patients, we cultured lesion swab samples from involved areas on nonselective media: blood agar plates (aerobic atmosphere) and chocolate agar plates (with 5% CO_2_). After 40 hours, cultures yielded β-hemolytic, rhizoid, adherent, rough, shiny, and yellowish colonies on both culture media (Figure 2, panels A, B), and occasionally along with colonies from the skin microbiota. Gram staining revealed identical filamentous, branching gram-positive bacilli with transverse and longitudinal septations producing a characteristic tram-track appearance (Figure 2, panel C). Bacterial identification by matrix-assisted laser desorption/ionization time-of-flight mass spectrometry identified *D. congolensis* with a high confidence score in all cases. In 4 patients, bacterial cultures also yielded pyogenic pathogens: *Staphylococcus aureus* (patients 3, 7 and 9) or *S. lugdunensis* (patient 8). Whole-genome sequencing (NextSeq550; Illumina, https://www.illumina.com) and pairwise alignment of *D. congolensis* isolates from patients 1–8 revealed 1–5 single-nucleotide polymorphisms, covering >94% of the genome, supporting close relatedness and recent direct or indirect transmission from a common source. Reads are available for consultation on the European Nucleotide Archive database (Table). Concomitant STIs were diagnosed in 2 patients (pubic pthiriasis in patient 1 and syphilitic reinfection in patient 7).

Epidemiologic interviews revealed that 7 of the 9 patients reported recent sexual encounters at a gay sauna in Lyon within days or weeks before symptom onset (Table). Patient 5 reported multiple sexual partners in various saunas in Paris during the same period, including 1 that patient 3 visited 2 days earlier. Patient 9 did not report any sauna attendance. On the basis of reported exposures, incubation period ranged from 3 to 14 days; survival modeling with interval censoring estimated a median of 6.7 (95% CI 4.3–10.1) days. However, multiple potential contacts limited precision (Table).

Amoxicillin MIC determined for 1 isolate using Etest (bioMérieux, https://www.biomerieux.com) was 0.064 mg/L, supporting high susceptibility to β-lactams. All patients received oral amoxicillin (1 g 3×/d) or pristinamycin (1 g 3×/d) for 7 days, sometimes combined with topical antiseptic care, with rapid improvement. No patient relapsed; median follow-up was 10 (IQR 5–52) days after treatment. However, on follow-up, patient 1 showed with a new *D. congolensis* infection on the buttocks, occurring 8 weeks after the first occurrence (Figure 1, panel D). His ongoing visits to the same sauna after recovery and the different infection site suggest reinfection rather than relapse.

Compared with previous reports of dermatophilosis, the predominantly papular and noncrusted manifestation observed here might differ from the classical description, raising the possibility of a distinct clinical phenotype. Moreover, lesions mainly affected areas exposed during sexual intercourse (face and genitals), mirroring patterns seen in sexually transmitted dermatophytosis or *K. aerogenes* folliculitis (*12**–**14*). Whereas no mucosal lesions were observed in this cluster, rare literature reports indicate that *D. congolensis* can infect human mucosa, justifying detailed clinical examination (*6*,*11*). In some occurrences, *S. aureus* and *S. lugdunensis* were also isolated from lesion sampling and might have modified clinical manifestations. Their involvement complicates the interpretation of *D. congolensis* pathogenicity in the given occurrences. Secondary infection of *Dermatophilus*-induced lesions by such pathogens is the most probable explanation, although co-transmission remains possible.

Although no direct sexual contact between patients could be formally established, the temporal clustering, overlapping sexual exposures, shared multiple STI history, lesion distribution, and close genomic relatedness of isolates strongly support transmission occurring within shared exposure networks, likely involving close physical or sexual contact. The presence of viable bacteria within lesions is consistent with the hypothesis of contact-driven transmission, although the exact transmission route remains uncertain. Environmental or indirect transmission within shared venues remains possible; no environmental sampling or carriage screening was performed at the time of the study to clarify transmission pathways.

## Conclusions

We describe a large occurrence of human dermatophilosis cases in an urban population of sexually active MSM without reported livestock exposure. The combination of close genomic relatedness between the 8 sequenced isolates and shared sexual exposures suggest interhuman transmission within sexual networks. Systematic microbiologic evaluation of atypical cutaneous lesions is essential for identifying similar cases and clarifying transmission of emerging cutaneous STIs among MSM. Evolving sexual practices in the HIV preexposure prophylaxis era (*15*) could lead to emergence of new transmissible dermatoses (*12**–**14*). Microbiologists should ensure that *D. congolensis* is included in reference spectral libraries, be able to recognize its colonies in the context of skin infection, and report its identification to clinicians.
